# Factors associated with severe neurologic complications in patients with either hand-foot-mouth disease or herpangina: A nationwide observational study in South Korea, 2009-2014

**DOI:** 10.1371/journal.pone.0201726

**Published:** 2018-08-10

**Authors:** Bongyoung Kim, Shinje Moon, Geun-Ryang Bae, Hyungmin Lee, Hyunjoo Pai, Sung Hee Oh

**Affiliations:** 1 Department of Internal Medicine, Hanyang University College of Medicine, Seoul, South Korea; 2 Department of Epidemic Intelligence Service, Korea Centers for Disease Control and Prevention, Osong, Cheongju, South Korea; 3 Department of Internal Medicine, Hallym University College of Medicine, Chuncheon, South Korea; 4 National Radiation Emergency Medical Center, Korea Institute of Radiological and Medical Sciences, Seoul, South Korea; 5 Department of Healthcare Associated Infection Control, Korea Centers for Disease Control and Prevention, Osong, Cheongju, South Korea; 6 Department of Pediatrics, Hanyang University College of Medicine, Seoul, South Korea; The University of Hong Kong, CHINA

## Abstract

**Background:**

In 2009, a nationwide sentinel surveillance for hand-foot-mouth disease (HFMD) and herpangina (HA) with neurologic complications was initiated in South Korea. We used this surveillance system to investigate the clinical characteristics of patients with either HFMD or HA with neurologic complications, with the aim of determining risk factors for severe neurologic complications.

**Methods:**

A retrospective review of medical records was conducted on all cases of HFMD and HA with neurologic complications that were reported in the national system between April 1, 2009 and December 31, 2014. A severe case was defined as having HFMD or HA with encephalitis, polio-like syndrome, or cardiopulmonary failure, and less-severe cases were defined as having HFMD or HA with aseptic meningitis.

**Results:**

A total of 138 cases (less-severe: 90/138, 65.2%; severe: 48/138, 24.8%) were included from 28 hospitals; 28 ineligible cases were excluded. Of 48 severe cases, 27 (56.2%) had encephalitis; 14 (29.2%) had polio-like syndrome; and seven (14.6%) had cardiopulmonary syndrome. The median patient age was 36 months (IQR: 18–60) and 63 (45.7%) patients were female. Most patients completely recovered, except for seven cases that were fatal or resulted in long-term symptoms (5.1%, 3 patients with neurologic sequelae and 4 deaths). In a multivariable logistic regression analysis, lethargy (OR = 4.67, 95% CI: 1.37–15.96, *P* = 0.014), female sex (OR = 3.51, 95% CI: 1.17–10.50, *P* = 0.025), and enterovirus A71 (OR = 3.55, 95% CI: 1.09–11.57, *P* = 0.035) were significantly associated with severe neurologic complications in HFMD and HA patients.

**Conclusion:**

In patients with HFMD and HA, lethargy, female, and enterovirus A71 may predict severe neurologic complications.

## Introduction

Hand-foot-mouth disease (HFMD) is a common viral infection syndrome in children that is characterized by macular, maculopapular, or vesicular rash on the hands, feet, mouth, buttocks, and other possible locations. Herpangina (HA) is characterized by fever and painful vesiculo-ulcerative oral lesions, without an accompanying skin rash [1]. Both HFMD and HA are caused by various enterovirus serotypes, with coxsackievirus A16 and enterovirus A71 (EV-A71) being the most common causative viral strains [1].

Although the majority of patients with HFMD or HA show a mild and self-limiting clinical course, some cases caused by EV-A71 tend to be more severe and even fatal. The cases caused by EV-A71 have relatively high percentage of neurological involvement throughout the course of illness, such as meningitis, encephalitis, and neurogenic pulmonary edema or hemorrhage [2].

In the late 1990s, EV-A71-associated HFMD and HA outbreaks occurred in several countries in the Asia-Pacific region, including Malaysia, Taiwan, and China; these outbreaks resulted in numerous deaths due to severe complications [3–5]. Several years later, South Korea also experienced an EV-A71-associated HFMD and HA epidemic, with multiple deaths occurring in 2009 [6–7]. Accordingly, the need for controlling neurologically-involved HFMD or HA became apparent, and the Korean Centers for Disease Control and Prevention (KCDC) designated both as national notifiable infectious diseases.

A previous study in Korea reported that headache and the presence of neurologic signs were significant risk factors for neurologic complications in HFMD or HA [1]. However, most aseptic meningitis usually presents with a mild and reversible clinical course, while encephalitis, polio-like syndrome, and/or cardiopulmonary failure can result in grave consequences, such as permanent neurological sequelae or death [8]. Therefore, investigations regarding factors associated with severe neurologic involvement are necessary. Thus, the aim of this study was to determine risk factors for severe neurologic complications in patients with either HFMD or HA, using data from Korea’s National Sentinel Surveillance System for HFMD with neurologic complications (NSSSH).

## Material and methods

### Study setting

Both HFMD and HA with neurologic complications were designated as national notifiable infectious diseases in 2009, and the NSSSH was established in the same year. All suspected cases diagnosed in tertiary care hospitals (a total of 44 tertiary care hospitals located throughout South Korea) are mandatorily reported by physicians to the KCDC through this surveillance system and cases were reported from 28 hospitals during 2009–2014. We retrospectively reviewed the medical records of all cases reported to this system between April 1, 2009 and December 31, 2014, gathering the following patient data: i) demographic data, ii) perinatal history, iii) clinical features and outcomes, iv) initial laboratory findings, and v) findings on magnetic resonance imaging (MRI). The retrospective review was performed by two investigators in KCDC from November 25, 2014 to February 17, 2015.

The total number of patients with HFMD and HA was collected from the Healthcare Big Data Hub of Health Insurance Review & Assessment Service; data were available from 2010–2014 [9].

To examine virology, we correlated our data with the data of the Nationwide Surveillance System for Infection with Enterovirus; this is another nationwide surveillance system. Via this system, the sentinel hospitals voluntarily sent specimens collected from patients with highly suspicious symptoms related to enterovirus infection including HFMD and HA to KCDC. The specimens were collected without consideration of the date of symptom onset. Once KCDC received samples, reverse transcription-PCR (RT-PCR) using TaqMan technology was applied to all stool specimens, throat swabs, and CSF samples in order to detect the enteroviral genome. Detailed information of this process has been described in a previous report [7]. For cases without having the viral test result at the Nationwide Surveillance System for Infection with Enterovirus, we collected enterovirus PCR results conducted at each hospital.

All collected data were anonymized before analysis.

### Case definitions

HFMD was defined as presence of vesiculopapular rashes in at least two classical anatomical sites, namely the hands, feet, buttocks, or mouth. HA was defined as presence of mouth ulcers without any skin rash. Aseptic meningitis was defined as the presence of pleocytosis (leukocyte >5 cell/μL) in cerebrospinal fluid (CSF) analysis, with a negative CSF bacterial culture result. Encephalitis was defined as pleocytosis (leukocyte >5 cell/μL) in CSF analysis, with a negative CSF bacterial culture result, and the presence of an altered level of consciousness and/or other neurologic signs. Polio-like syndrome was characterized by the acute onset of areflexic limb weakness and/or decreased deep tendon reflex. Cardiopulmonary syndrome was defined as the presence of respiratory distress, pulmonary edema, and pulmonary congestion on chest radiograph or symptoms and/or signs compatible with myocarditis.

We defined “less-severe” cases as cases of HFMD or HA with aseptic meningitis; “severe” cases had HFMD or HA with at least one of the following: encephalitis, polio-like syndrome, or cardiopulmonary syndrome. We excluded any cases that did not meet the case definition of either less-severe or severe.

### Statistical analysis

All statistical analyses were performed using SPSS version 21.0 for Windows (IBM Corporation, Armonk, NY, USA). Categorical variables were analyzed by the chi-square test or Fisher’s exact test. Continuous variables were analyzed using the Mann–Whitney U test or the independent *t*-test. A multivariable logistic regression analysis was performed to evaluate the effect of independent variables on risk. In order to avoid collinearity among the variables, we adopted a stepwise regression method with backward elimination. Using a two-tailed test, a *P*-value of < 0.05 was considered to be statistically significant.

### Ethics statement

The study protocol was approved by the institutional review boards of KCDC (IRB number: 2014-12EXP-02-P-E), and the requirement for written informed consent from patients was waived.

## Results

### Demographic characteristics and clinical manifestations

**Fig 1:**
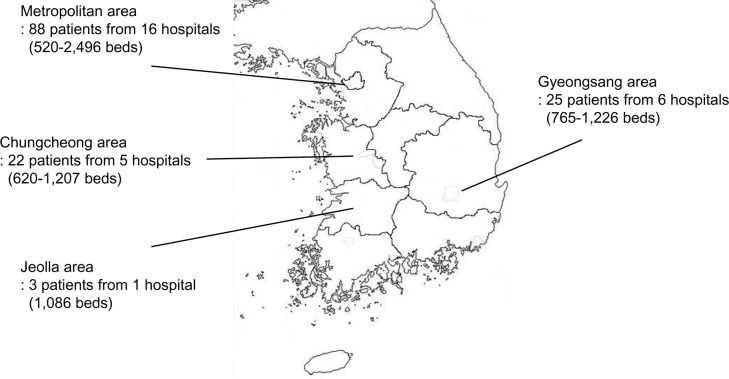
Geographic distribution of tertiary care hospitals that has reported cases of hand-foot-mouth disease or herpangina with neurologic complications in South Korea, 2009–2014.

**Fig 2:**
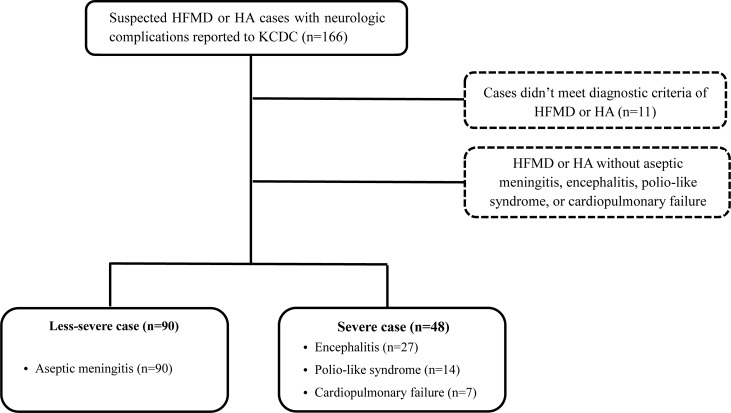
Flow diagram showing the process for selecting cases in this study. Abbreviations: HFMD, hand-foot-mouth disease; HA, herpangina; KCDC, Korean Centers for Disease Control and Prevention.

A total of 138 cases were included from 28 tertiary care hospitals (520–2,496 beds) throughout the Korean peninsula (Fig 1), and data of 28 patients were excluded: 11 cases did not meet the diagnostic criteria of HFMD or HA, and 17 cases did not meet the case definition of severe or less-severe. Of the 138 included cases, 128 (92.8%) had HFMD and 10 (7.2%) had HA. The majority of cases were classified as less-severe (65.2%, 90/138), while 48 (34.8%) cases were classified as severe. Among the severe cases, 27 (56.2%) patients presented with encephalitis; 14 (29.2%) with polio-like syndrome; and seven (14.6%) with cardiopulmonary syndrome (Fig 2).

**Fig 3:**
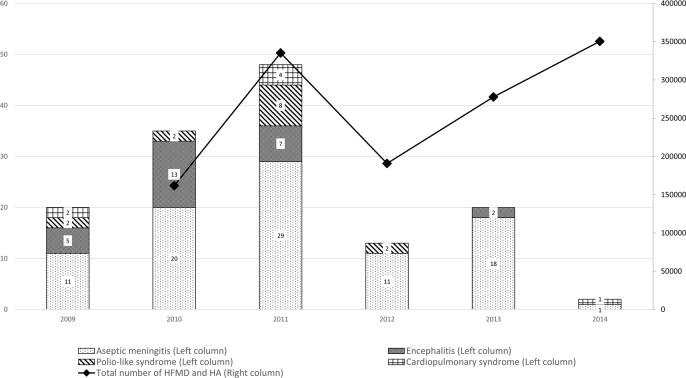
The annual number of reported cases of hand-foot-mouth disease or herpangina with neurologic complications in South Korea, 2009–2014. Abbreviations: HFMD, hand-foot-mouth disease; HA, herpangina.

The number of patients with either HFMD or HA and neurologic complications increased gradually from 2009 and showed peak incidence in 2011. The annual number of reported cases decreased thereafter and only two cases were reported in 2014. However, the total number of patients with either HFMD or HA regardless of neurologic complication decreased from 2011 to 2012, but slightly increased in 2013 (Fig 3).

Viral studies were performed in 121 cases (87.7%, 121/138): 71 results were obtained from the Nationwide Surveillance System for Infection with Enterovirus and 50 results were obtained from medical records in each hospital. Of these 121 cases, enterovirus was detected in 89 (73.6%) patients. EV-A71 (80.9%, 72/89) was the most common virus serotype, followed by coxsackievirus A16 (3.4%, 3/89), coxsackievirus B4 (2.2%, 2/89), and echovirus 9 (1.1%, 1/89), as well as unidentified viral types in 11 cases (12.4%, 11/89).

The overall clinical characteristics of the study participants are shown in Table 1. The ratio of HFMD to HA was approximately 13:1. The median patient age was 36 months (interquartile range [IQR]: 18–60) and 63 (45.7%) patients were female. The median patient height was 97.7 cm (IQR: 83.0–112.6) and the median weight was 14.1 kg (IQR: 11.2–19.1). The ratio of normal spontaneous vaginal delivery to cesarean section was 2:1 (81:38). The mean patient birth weight was 3.37 ± 0.49 kg and the mean gestational period was 39.0 ± 1.5 weeks.

**Table 1:** Clinical characteristics of patients with hand-foot-mouth disease or herpangina with neurologic complications.

	Less-severe case (n = 90)	Severe case (n = 48)	*P*-value	Total (n = 138)
Virology (n = 121)				
**Enterovirus A71 (%)**	**38 (50)**	**34 (75.6)**	**0.006**	**72 (59.5)**
Clinical category				
Hand-foot-mouth disease (%)	83 (92.2)	45 (93.8)	1.000	128 (92.8)
Herpangina (%)	7 (7.8)	3 (6.2)	1.000	10 (7.2)
Encephalitis (%)	-	27 (56.2)	-	27 (19.6)
Polio-like syndrome (%)	-	14 (29.2)	-	14 (10.1)
Cardiopulmonary syndrome (%)	-	7 (14.6)	-	7 (5.1)
Demographic data				
Age (months)^**1**^	39 (15–63)	28 (19–48)	0.196	36 (18–60)
**Female sex (%)**	**35 (38.9)**	**28 (58.3)**	**0.030**	**63 (45.7)**
Height (cm)^**1**^	103.0 (83.0–114.5)	93.0 (83.0–109.5)	0.373	97.7 (83–112)
Weight (kg)^**1**^	14.9 (10.5–19.5)	13.6 (11.4–18.0)	0.590	14.1 (11.2–18.8)
Perinatal history				
Types of Delivery (NSVD/C-Sec) (%)	50/28 (64.1/35.9)	31/10 (75.6/24.4)	0.203	81/38 (68.1/31.9)
Birth weight (kg), mean ± SD	3.32 ± 0.43	3.45 ± 0.57	0.187	3.37 ± 0.49
Gestational period (weeks), mean ± SD	39.00 ± 1.56	39.08 ± 1.40	0.797	39.03 ± 1.50
Clinical features				
Days before hospitalization, mean ± SD	3.28 ± 1.94	3.69 ± 2.71	0.308	3.42 ± 2.24
Fever (≥38°C) (%)	78 (86.7)	37 (77.1)	0.150	115 (83.3)
Total febrile duration (days), mean ± SD	2.33 ± 1.51	2.94 ± 4.48	0.369	2.54 ± 2.91
**Lethargy (%)**	**15 (16.7)**	**19 (39.6)**	**0.004**	**34 (24.6)**
Cough (%)	17 (18.9)	11 (22.9)	0.575	28 (20.3)
Rhinorrhea (%)	19 (21.1)	10 (20.8)	0.970	29 (21.0)
Poor oral intake (%)	50 (55.6)	29 (60.4)	0.582	79 (57.2)
Nausea (%)	51 (56.7)	30 (62.5)	0.507	81 (58.7)
Vomiting (%)	55 (61.1)	33 (68.8)	0.374	88 (63.8)
Abdominal pain (%)	11 (12.2)	5 (10.4)	0.752	16 (11.6)
Diarrhea (%)	2 (2.2)	1 (2.1)	1.000	3 (2.2)
Dizziness (%)	9 (10.0)	5 (10.4)	1.000	14 (10.1)
Rash location^2^				
Hands (%)	75 (83.3)	35 (72.9)	0.147	110 (79.7)
Feet (%)	74 (82.2)	33 (68.8)	0.071	107 (77.5)
Mouth (%)	65 (72.2)	32 (66.7)	0.496	97 (70.3)
Buttocks (%)	16 (17.8)	7 (14.6)	0.632	23 (16.7)
**Clinical outcomes**				
**Hospitalization days**^**1**^	**6 (5–7)**	**11 (7–13)**	**<0.001**	**6 (4–9)**
**Recovery (%)**	**90 (100)**	**41 (85.4)**	**<0.001**	**131 (94.9)**
**Neurologic sequelae (%)**	**0 (0)**	**3 (6.3)**	**-**	**3 (2.2)**
**Death (%)**	**0 (0)**	**4 (8.3)**	**-**	**4 (2.9)**

Abbreviations: NSVD, Normal spontaneous vaginal delivery; C-sec, Cesarean section; SD, Standard deviation.

^1^; These data are shown as median (25–75 percentile range).

^2^; Multiple responses.

Among clinical features, the most common symptom was fever (83.3%, 115/138), with a total duration of 2.54 ± 2.91 days. Other common symptoms were vomiting (63.8%, 88/138), nausea (58.7%, 81/138), and poor oral intake (57.2%, 79/138). When cases were analyzed according to rash location, the most frequently involved areas were the hands (79.7%, 110/138), followed by the feet (77.5%, 107/138), mouth (70.3%, 97/138), and buttocks (16.7%, 23/138). Forty-eight (34.8%) patients had erythematous lesions, 15 (10.9%) had vesicular lesions, and 18 (13.0%) had ulcerative lesions.

### Comparison of clinical characteristics

Among virus serotypes, the proportion of EV-A71 was significantly higher in severe cases compared to less-severe cases (less-severe: 50%, 38/76; severe: 75.6%, 34/45; respectively, *P* = 0.006). The ratio of HFMD to HA was similar in both groups (*P* = 1.000). The median age of less-severe and severe cases was 39 months (IQR: 15–63) and 28 months (IQR: 19–48), respectively (*P* = 0.196). Female sex predominated in severe cases than in less-severe cases (less severe: 38.9%, 35/90; severe: 58.3%, 28/48; respectively, *P* = 0.030). In both groups, the majority of patients were birthed naturally (less-severe: 64.1%, 50/90; severe: 75.6%, 31/48; respectively, *P* = 0.203). The mean birth weight was 3.32 ± 0.43 kg in less-severe cases and 3.45 ± 0.57 kg in severe cases (*P* = 0.187); the mean gestational period was 39.00 ± 1.56 weeks in less-severe cases and 39.08 ± 1.40 weeks in severe cases (*P* = 0.797). Lethargy was less frequent in less-severe cases than in severe cases (less severe: 16.7%, 15/90; severe: 39.6%, 19/48; respectively, *P* = 0.004). There were no significant differences in rash location or type between the two groups.

In terms of clinical outcomes, median hospitalization duration was longer in severe cases compared with less-severe cases (less severe: 6 days; severe: 11 days; respectively, *P* < 0.001). Most patients completely recovered, except for seven cases that were fatal or resulted in long-term symptoms (5.1%, 7/138; 3 patients with neurologic sequelae and 4 deaths). All seven cases occurred in the group of severe case; all fatal cases were from the group of severe case and had cardiopulmonary syndrome (57.1%, 4/7). Each of the three patients survived with neurologic sequelae had encephalitis, polio-like syndrome, or cardiopulmonary syndrome, respectively.

### Comparison of initial laboratory findings

The overall initial laboratory findings of the two groups are shown in Table 2. There were no significant differences in CSF analyses between the two groups. White blood cell (WBC) count was lower in less-severe cases compared to severe cases (less-severe: 11,282.2 ± 4,520.6; severe: 13,184.8 ± 5,107.6; respectively, *P* = 0.030), as were erythrocyte sedimentation rates (less-severe: 20.3 ± 16.5; severe: 32.0 ± 25.4; respectively, *P* = 0.011); additionally, the less-severe cases had higher phosphate levels than did severe cases (less severe: 4.7 ± 0.8; severe: 4.3 ± 1.0; respectively, *P* = 0.016), and higher albumin levels (less-severe: 4.5 ± 0.4; severe: 4.2 ± 0.5; respectively, *P* = 0.004).

**Table 2:** Initial laboratory findings of patients with Hand-foot-mouth disease or herpangina with neurologic complications.

	Less-severe case (n = 90)	Severe case (n = 48)	*P*-value
WBC count (CSF) (cells/mm^3^)	182.2 ± 240.9	165.7 ± 155.6	0.745
Protein (CSF) (mg/dL)	48.3 ± 36.0	51.2 ± 35.8	0.663
Glucose (CSF) (mg/dL)	65.7 ± 11.5	69.5 ± 12.2	0.077
**WBC count (cells/mm**^**3**^**)**	**11,282.2 ± 4,520.6**	**13,184.8 ± 5,107.6**	**0.030**
Hemoglobin (g/dL)	13.5 ± 10.6	14.6 ± 17.2	0.626
Platelet count (cells^10^3^/mm^3^)	323.5 ± 90.8	332.6 ± 89.1	0.570
**Erythrocyte sedimentation rate (mm/hr)**	**20.3 ± 16.5**	**32.0 ± 25.4**	**0.011**
C-reactive protein (mg/dL)	2.3 ± 4.9	5.8 ± 14.6	0.117
Glucose (mg/dL)	105.9 ± 23.2	111.8 ± 35.6	0.244
Calcium (mg/dL)	9.8 ± 0.4	9.7 ± 0.7	0.161
**Phosphate (mg/dL)**	**4.7 ± 0.8**	**4.3 ± 1.0**	**0.016**
Protein (g/dL)	7.1 ± 0.7	7.0 ± 0.7	0.287
**Albumin (g/dL)**	**4.5 ± 0.4**	**4.2 ± 0.5**	**0.004**
Lactate dehydrogenase (U/L)	472.2 ± 211.7	497.1 ± 209.5	0.658

Abbreviations: WBC, White blood cell; CSF, Cerebrospinal fluid.

Note: These data are shown as mean **±** standard deviation.

### Neurologic findings in severe case

Table 3 shows neurologic findings in patients with HFMD or HA with severe neurologic complications. Seizure (39.6%, 19/48) was the most common neurologic symptom, followed by myoclonic jerks (29.2%, 14/48), ataxia (12.5%, 6/48), and nystagmus (10.4%, 5/48).

**Table 3:** Neurologic findings in patients with Hand-foot-mouth disease or herpangina with severe neurologic complications.

	Encephalitis	Polio-like syndrome	Cardiopulmonary syndrome	*P*-value	Total
Neurologic symptoms and signs					
Nystagmus (%)	5/27 (18.5)	0/14 (0)	0/7 (0)	0.114	5/48 (10.4)
Myoclonic jerks (%)	10/27 (37.0)	3/14 (21.4)	1/7 (14.3)	0.374	14/48 (29.2)
Dysphagia (%)	1/27 (3.7)	0/14 (0)	0/7 (0)	0.672	1/48 (2.1)
Dysarthria (%)	2/27 (7.4)	1/14 (7.1)	0/7 (0)	0.761	3/48 (6.3)
Ataxia (%)	3/27 (11.1)	3/14 (21.4)	0/7 (0)	0.356	6/48 (12.5)
Neurogenic bladder (%)	0/27 (0)	1/14 (7.1)	0/7 (0)	0.289	1/48 (2.1)
Seizure (%)	10/27 (37.0)	5/14 (35.7)	4/7 (57.1)	0.588	19/48 (39.6)
Abnormal findings in MRI by location					
Cerebrum (%)	3/11 (27.3)	1/9 (11.1)	1/1 (100)	0.131	5/21 (23.8)
Cerebellum (%)	2/11 (18.2)	1/9 (11.1)	0/1 (0)	0.828	3/21 (14.3)
Pons (%)	6/11 (54.5)	6 (66.7)	0/1 (0)	0.428	12/21 (57.1)
Medulla (%)	5/11 (45.4)	3/9 (33.3)	0/1 (0)	0.620	8/21 (38.1)
Midbrain (%)	2/11 (18.2)	0/9 (0)	0/1 (0)	0.366	2/21 (9.5)
**Spinal cord (%)**	**0/11 (0)**	**4/9 (44.4)**	**0/1 (0)**	**0.037**	**4/21 (19.0)**

Abbreviations: MRI, Magnetic resonance imaging.

MRI were performed for 21 patients in the group of severe case; these showed that the region most vulnerable to lesions was the pons (57.1%, 12/21), followed by the medulla (38.1%, 8/21), cerebrum (23.8%, 5/21), and spinal cord (19.0%, 4/21). There were no significant differences in most lesion locations among encephalitis, polio-like syndrome, and cardiopulmonary syndrome, with one exception: spinal cord lesions were predominant in patients with polio-like syndrome (encephalitis: 0%, 0/11; polio-like syndrome: 4/9, 44.4%; cardiopulmonary syndrome: 0/1, 0%; *P* < 0.001).

### Multivariate analysis of risk factors for HFMD or HA with severe neurologic complications

The relative risks, determined by multivariate analysis of all statistically significant variables from univariate analysis, are shown in Table 4. Lethargy (OR = 4.67, 95% CI: 1.37–15.96, *P* = 0.014), female sex (OR = 3.51, 95% CI: 1.17–10.50, *P* = 0.025), and EV-A71 (OR = 3.55, 95% CI: 1.09–11.57, *P* = 0.035) were significantly associated with both HFMD and HA that presented with severe neurologic complications.

**Table 4:** Risk factors for Hand-foot-mouth disease or herpangina with severe neurologic complications using a multivariable logistic regression model.

Predictors	No.	Univariate analysis		Multivariate analysis	
		OR (95% CI)	*P*-value	OR (95% CI)	*P*-value
Age <18 months					
No	104	1.0	-	-	-
Yes	34	1.38 (0.60–3.19)	0.450	-	-
**Female sex**					
No	75	1.0	-	1.0	-
Yes	63	2.20 (1.08–4.49)	0.030	**3.51 (1.17–10.50)**	**0.025**
**Enterovirus A71**				-	-
No	49	1.0	-	1.0	-
Yes	72	3.25 (1.44–7.33)	0.004	**3.55 (1.09–11.57)**	**0.035**
**Lethargy**					
No	106	1.0	-	1.0	-
Yes	32	3.28 (1.47–7.30)	0.004	**4.67 (1.37–15.96)**	**0.014**
WBC ≥15,000 cells/mm^3^					
No	107	1.0	-	-	-
Yes	30	1.95 (0.87–4.37)	0.104	**-**	**-**
ESR ≥20 mm/hr					
No	41	1.0	-	1.0	-
Yes	54	2.15 (0.92–5.02)	0.076	2.55 (0.92–7.06)	0.071
Phosphate ≥5.1 mg/dL					
No	95	1.0	-	1.0	-
Yes	33	0.50 (0.20–1.23)	0.132	0.32 (0.09–1.16)	0.082
Albumin <4.2 g/dL					
No	107	1.0	-	-	-
Yes	30	2.80 (1.22–6.43)	0.015	-	-

Abbreviations: OR, Odd ratio; CI, confidence interval; WBC, White blood cell; ESR, Erythrocyte sedimentation rate.

## Discussion

This is the first report to describe the clinical characteristics of HFMD or HA with neurologic complications using the NSSSH. In this nationwide study, the median age of patients was 36 months, which is relatively higher than that of patients with HFMD or HA in Malaysia (median, 23 months) [10] and China (mean, 2.17 ± 1.19 years) [11], but similar that in a previous study in South Korea (median, 33.5 months) and Japan (mean, 3.47 ± 2.41 years) [12]. We found that aseptic meningitis was the most common neurologic complication of HFMD and HA, throughout all study years. This finding is concordant with previous studies in Asia-Pacific region. The proportion of aseptic meningitis among cases of HFMD or HA with neurologic complications was 63.6% of 88 cases in a Korean study, approximately 80% of 199 cases in a Japanese study, and 84.7% of 333 cases in a Taiwanese study [1, 12, 13]. Additionally, the common symptoms of HFMD or HA with neurologic complications found in the present report were similar to findings of previous studies: two previous Korean studies reported that fever, vomiting, and headache were common symptoms [1, 7]. Similarly, according to a Chinese study, fever, vomiting, and hypersomnia were the most common symptoms for HFMD with neurologic complications [14].

Similar to the findings from a previous study [15], our findings showed that the pons and medulla were the most commonly-affected sites in the central nervous system. EV-A71 has a tissue tropism in the brain stem, cerebellum, and spinal cord anterior horn cell, and clinical manifestations may vary according to the affected site [16]; for example, when EV-A71 invades the anterior horn or root of the spinal cord, limb impairment may present [17]. The genogroups of EV-A71 may influence the risk of neurologic involvement; Ooi et al. emphasized that EV-A71 genogroup B4 was less likely to have neurologic complications compared with genogroup C1 or B5 [10]. In the present study, C4a was dominant genogroup (79.6%, 43/54) and higher proportion of severe case was observed in genogroup C4a compared with non-C4a (51.2%, 22/43; 9.1%, 1/11; respectively, *P* = 0.012) (S2 File). However, the preference to affected sites according to genogroups could not be analyzed due to lack of adequate sample size.

Previous studies have focused on analyzing the risk factors of fatal cases of HFMD and HA. Chong et al. compared seven fatal and 131 non-fatal HFMD cases and found that atypical physical findings, raised WBC count, vomiting, and the absence of mouth ulcers were predictive of a fatal disease progression [18]. A Chinese case-control study reported that EV-A71, convulsion, dyspnea, cyanosis, coolness of extremities, and vomiting were risk factors for death in children with HFMD [19]. In another Chinese observational study, female sex, light-reflex insensitivity, tachycardia, and higher serum lactate level were independent risk factors for fatality among patients with HFMD with neurologic complications [11]. According to Chang et al., hyperglycemia, leukocytosis, and limb weakness were prognostic factors for pulmonary edema after neurologic involvement among EV-A71-associated HFMD patients; additionally, the authors postulated that hyperglycemia might have resulted from autonomic nervous system dysfunction, which regulates blood glucose homeostasis in HFMD or HA with neurologic complications [20]. Pulmonary edema is the most fulminant complication associated with HFMD and HA, and is caused by the invasion of EV-A71 into the medullary vasomotor center [21].

There are a few studies focused on analyzing the risk factors of HFMD or HA with neurologic complications. A meta-analysis found that prolonged duration of fever (≥3 days), lethargy, hyperglycemia, vomiting, increased neutrophil count, EV-A71 infection, and younger age were significantly associated with severe HFMD including neurologic complications [22]. Furthermore, when the causative pathogen was confined to EV-A71, young age, fever, vomiting, mouth ulcers, breathlessness, cold limbs, and poor urine output were associated with neurologic complications [10]. Similarly, the results of the present study indicated that lethargy, female sex, and EV-A71 were the primary risk factors for HFMD or HA with severe neurologic complications.

There were some limitations to our study. Firstly, the NSSSH gathers data from tertiary care hospitals and cases diagnosed at other hospital types were not included; therefore, our results may not be generalizable. Secondly, certain number of patients may not have been included since the reporting to NSSSH was dependent on each physician’s compliance. Third, the NSSSH was established during the 2009 epidemic. Cases which occurred during the first quarter in 2009 were not included and the annual incidence in 2009 might be underestimated. Finally, severe cases were compared with less-severe cases, not with non-severe cases (HFMD or HA without neurologic complications), which may have led us to less precise comparison. Despite these limitations, the overall data of this study is likely to be a reasonable approximation of the true value, and our findings may represent the national status of HFMD or HA with neurologic complications in South Korea.

In conclusion, lethargy, female, and EV-A71 in patients with HFMD or HA may predict the development of severe neurologic complications and require early recognition and careful management for patients that exhibit these risk factors.

## Supporting information

S1 File The process of stepwise regression method with backward elimination for determining risk factors for hand-foot-mouth disease or herpangina with severe neurologic complications. (DOCX)

S2 File Comparison of clinical characteristics and neurologic findings of hand-foot-mouth disease or herpangina with neurologic complications according to genogroup of enterovirus. (DOCX)
