# Supplementary material for: Factors associated with severe neurologic complications in patients with either hand-foot-mouth disease or herpangina: A nationwide observational study in South Korea, 2009-2014
Source: PLoS One. 2018 Aug 10;13(8):e0201726. doi: 10.1371/journal.pone.0201726 (PMC6086402; doi:10.1371/journal.pone.0201726)
Supplement: S1 File — (DOCX) [file pone.0201726.s001.docx]

S1 Table. The process of stepwise regression method with backward elimination for determining risk factors for hand-foot-mouth disease or herpangina with severe neurologic complications.

|  | | *P*-value | Exp(B) | 95% Confidence interval | |
| --- | --- | --- | --- | --- | --- |
|  |  |  |  | Lower bound | Upper bound |
| Step 1 | Age <18 months | 0.423 | 0.562 | 0.137 | 2.304 |
|  | Female sex | 0.063 | 0.335 | 0.106 | 1.059 |
|  | Enterovirus A71 | 0.029 | 3.957 | 1.152 | 13.589 |
|  | Lethalgy | 0.090 | 3.082 | 0.839 | 11.315 |
|  | WBC ≥17500 cells/mm^3^ | 0.105 | 3.949 | 0.750 | 20.803 |
|  | ESR ≥20 mm/hr | 0.076 | 2.681 | 0.903 | 7.963 |
|  | Phosphate ≥5.1 mg/dL | 0.057 | 0.242 | 0.056 | 1.042 |
|  | Albumin <4.2 g/dL | 0.752 | 1.220 | 0.354 | 4.203 |
| Step 2 | Age <18 months | 0.369 | 0.534 | 0.136 | 2.101 |
|  | Female sex | 0.052 | 0.324 | 0.104 | 1.007 |
|  | Enterovirus A71 | 0.028 | 3.997 | 1.166 | 13.703 |
|  | Lethalgy | 0.082 | 3.145 | 0.863 | 11.463 |
|  | WBC ≥17500 cells/mm^3^ | 0.106 | 3.893 | 0.749 | 20.231 |
|  | ESR ≥20 mm/hr | 0.065 | 2.752 | 0.938 | 8.072 |
|  | Phosphate ≥5.1 mg/dL | 0.058 | 0.247 | 0.058 | 1.050 |
| Step 3 | Female sex | 0.032 | 0.295 | 0.097 | 0.901 |
|  | Enterovirus A71 | 0.025 | 4.076 | 1.191 | 13.947 |
|  | Lethalgy | 0.070 | 3.298 | 0.909 | 11.968 |
|  | WBC ≥17500 cells/mm^3^ | 0.122 | 3.505 | 0.716 | 17.163 |
|  | ESR ≥20 mm/hr | 0.090 | 2.460 | 0.870 | 6.951 |
|  | Phosphate ≥5.1 mg/dL | 0.085 | 0.329 | 0.093 | 1.168 |
| Step 4 | Female sex | 0.025 | 0.285 | 0.095 | 0.852 |
|  | Enterovirus A71 | 0.035 | 3.555 | 1.092 | 11.568 |
|  | Lethalgy | 0.014 | 4.670 | 1.366 | 15.961 |
|  | ESR ≥20 mm/hr | 0.071 | 2.553 | 0.923 | 7.057 |
|  | Phosphate ≥5.1 mg/dL | 0.082 | 0.323 | 0.090 | 1.157 |

Abbreviations: WBC, White blood cell; ESR, Erythrocyte sedimentation rate
