# Supplementary material for: Factors associated with severe neurologic complications in patients with either hand-foot-mouth disease or herpangina: A nationwide observational study in South Korea, 2009-2014
Source: PLoS One. 2018 Aug 10;13(8):e0201726. doi: 10.1371/journal.pone.0201726 (PMC6086402; doi:10.1371/journal.pone.0201726)
Supplement: S2 File — (DOCX) [file pone.0201726.s002.docx]

Table S2. Comparison of clinical characteristics and neurologic findings of hand-foot-mouth disease or herpangina with neurologic complications according to genogroup of enterovirus.

|  | C4a (n=43) | Non-C4a (n=11) | *P*-value |
| --- | --- | --- | --- |
| Clinical category |  |  |  |
| **Less-severe case** | **21 (48.8)** | **10 (90.9)** | **0.012** |
| **Severe case** | **22 (51.2)** | **1 (9.1)** | **-** |
| Demographic data |  |  |  |
| Age (months)^1^ | 28 (12-51) | 42 (20-74) | 0.183 |
| Female sex (%) | 16 (37.2) | 5 (45.5) | 0.617 |
| Clinical features |  |  |  |
| Days before hospitalization, mean±SD | 3.56±2.26 | 2.73±0.90 | 0.240 |
| Fever (≥38℃) (%) | 36 (83.7) | 8 (72.7) | 0.408 |
| Total febrile duration (days), mean±SD | 2.35±2.12 | 2.36±1.80 | 0.983 |
| Lethargy (%) | 13 (30.2) | 3 (27.3) | 1.000 |
| Cough (%) | 12 (27.9) | 1 (9.1) | 0.261 |
| Rhinorrhea (%) | 11 (25.6) | 1 (9.1) | 0.421 |
| Poor oral intake (%) | 24 (55.8) | 9 (81.8) | 0.170 |
| Nausea (%) | 27 (62.8) | 7 (63.6) | 1.000 |
| Vomiting (%) | 28 (65.1) | 7 (63.6) | 1.000 |
| Abdominal pain (%) | 2 (4.7) | 1 (9.1) | 0.502 |
| Diarrhea (%) | 2 (4.7) | 0 (0) | 1.000 |
| **Dizziness (%)** | **2 (4.7)** | **4 (36.4)** | **0.012** |
| Neurologic symptoms and signs |  |  |  |
| Nystagmus (%) | 1 (2.3) | 1 (9.1) | 0.369 |
| Jerking (%) | 7 (16.3) | 1 (9.1) | 1.000 |
| Dysphagia (%) | 0 (0) | 0 (0) | - |
| Dysarthria (%) | 0 (0) | 0 (0) | - |
| Ataxia (%) | 2 (4.7) | 0 (0) | 1.000 |
| Neurogenic bladder (%) | 0 (0) | 0 (0) | - |
| **Seizure (%)** | **13 (30.2)** | **0 (0)** | **0.048** |
| Abnormal findings in MRI by location |  |  |  |
| Cerebrum (%) | 2/8 (25.0) | - | - |
| Cerebellum (%) | 1/8 (12.5) | - | - |
| Pons (%) | 5/8 (62.5) | - | - |
| Medulla (%) | 4/8 (50.0 | - | - |
| Midbrain (%) | 2/8 (25.0) | - | - |
| Spinal cord (%) | 0/8 (0) | - | - |
| Clinical outcomes |  |  |  |
| Hospitalization days^1^ | 8 (6-12) | 6 (5-8) | 0.112 |
| Recovery (%) | 39 (90.7) | 11 (100) | 0.571 |
| Neurologic sequele (%) | 0 (0) | 0 (0) | - |
| Death (%) | 4 (9.3) | 0 (0) | - |

^1^; These data are shown as median (25-75 percentile range)
